# Twentieth Century Practice

**Published:** 1898-09

**Authors:** 


TWENTIETH CENTURY PRACTICE.—An International Encyclopedia of Modern Medical Science. By leading authorities of Europe and America. Edited by Thomas L. Stedman, M. D., New York City. In twenty volumes. Volume XIV, "Infectious Diseases." New York: William Wood & Company. 1898.

For the use of the general practitioner this volume is perhaps the most important one that has yet been issued of this work. The first subject treated of is scarlet fever, by Dr. F. Forcheimer, of Cincinnati. Dr. Forcheimer treats the subject at great length, his article covering more than one hundred pages of the volume, and it is hardly worth the while to say that it is a most valuable contribution to the medical literature on this most important subject.

Dr. Dawson Williams, of London, contributes an interesting article on measles, in which he devotes much space to the consideration of the complications of measles.

In an article on German measles, Dr. F. Forcheimer agrees with Griffith that the term "rotheln" should never be used in this country because it is not English. He contends that if we are to use a foreign term it should be one of a universal language, which in our profession is the Latin.

Dr. Dillon Brown, of New York, writes the chapter on chickenpox, and Dr. Dawson Williams, of London, one on glandular fever. Whooping-cough is the subject of an article by Drs. Joseph O'Dwyer and Nathaniel Read Norton, of New York. This is probably the last of Dr. O'Dwyer's contributions to medical literature before his death.

A most instructive article is the one on cholera infantum by Dr. A. Jacobi, of New York.

Cholera Notras is the subject of an exhaustive article by Dr. Theodor Rumpf, of Hamburg. Dengue is discussed by Sir Joseph Fayrer, of London, and beriberi by Dr. A. A. de Aze vedo Sodre, of Rio de Janeiro.

The chapter on milliary fever was contributed by Dr. A. Netter, of Paris, and the closing chapter, on malta fever, by Dr. David Bruce of the British army. The most of the subjects discussed in this volume are of the greatest interest to the physicians of this country, since they treat of diseases which are frequently encountered. The volume, as a whole, is one of unusual merit and importance. S. E. H.

